# Quantification of Blood Caffeine Levels in Patients With Parkinson's Disease and Multiple System Atrophy by Caffeine ELISA

**DOI:** 10.3389/fneur.2020.580127

**Published:** 2020-12-22

**Authors:** Takuma Ohmichi, Takashi Kasai, Makiko Shinomoto, Jun Matsuura, Takashi Koizumi, Fukiko Kitani-Morii, Harutsugu Tatebe, Hidenao Sasaki, Toshiki Mizuno, Takahiko Tokuda

**Affiliations:** ^1^Department of Neurology, Kyoto Prefectural University of Medicine, Kyoto, Japan; ^2^Department of Functional Brain Imaging Research, National Institute of Radiological Sciences, National Institutes for Quantum and Radiological Science and Technology, Chiba, Japan; ^3^Department of Neurology, Faculty of Medicine and Graduate School of Medicine, Hokkaido University, Sapporo, Japan; ^4^AMED (Japan Agency for Medical Research and Development)-CREST, Tokyo, Japan

**Keywords:** caffeine, biomarkers, Parkinson's disease, multiple system atrophy, ELISA

## Abstract

Caffeine is considered to be a neuroprotective agent against Parkinson's disease (PD) and is expected to offer a blood-based biomarker for the disease. We herein investigated the ability of this biomarker to discriminate between PD and neurodegenerative diseases. To quantify caffeine concentrations in serum and plasma, we developed a specific competitive enzyme-linked immunosorbent assay (ELISA). To validate the diagnostic performance of the assay, we conducted a case control-study of two independent cohorts among controls and patients with PD and multiple system atrophy (MSA). Parallelism, recovery rate, and intra- and inter-assay precision of our assay were within the standard of acceptance. In the first cohort of 31 PD patients, 18 MSA patients and 33 age-matched controls, serum caffeine levels were significantly lower in PD patients than in Controls (*p* = 0.018). A similar trend was also observed in the MSA group, but did not reach the level of significance. In the second cohort of 50 PD patients, 50 MSA patients and 45 age-matched controls, plasma caffeine levels were significantly decreased in both PD and MSA groups compared to Controls (*p* < 0.001). This originally developed ELISA offered sufficient sensitivity to detect caffeine in human serum and plasma. We reproducibly confirmed decreased blood concentrations of caffeine in PD compared to controls using this ELISA. A similar trend was observed in the MSA group, despite a lack of consistent significant differences across cohorts.

## Introduction

Many reports have examined the relationship between Parkinson's disease (PD) and caffeine in epidemiology, animal experiments, and clinical pharmacology. Epidemiologically, caffeine intake has been established to exert neuroprotective effects against onset and progression of PD (1–4). In a PD animal model treated with 1-methyl-4-phenyl-1,2,3,6-tetrahydropyridine, caffeine attenuates the degeneration of dopaminergic neurons by inhibiting the adenosine A2A receptor (5). Based on clinical trial results, a selective adenosine A2A receptor antagonist of istradefylline improved motor symptoms of patients with PD (6). Recent metabolome analyses have shown that blood levels of caffeine metabolites were significantly lower in PD patients than in healthy subjects, and could be used as a candidate biomarker for predicting progression of PD (7–9).

In those reports, however, plasma caffeine concentration was quantified using mass spectrometry, which is ill-suited to application in clinical practice due to the high running costs. For clinical usage, cost-effective measurements of molecule concentration using an apparatus capable of easy maintenance, such as enzyme-linked immunosorbent assay (ELISA), still need to be developed. Moreover, the utility of quantifying caffeine concentration has not been confirmed as useful for discriminating PD from other neurodegenerative diseases, such as multiple system atrophy (MSA).

This study aimed to develop a novel caffeine ELISA applicable to human plasma/serum and to validate the method. We then conducted case-control studies of two independent cohorts to compare plasma and serum caffeine concentrations among control participants and individuals with PD and MSA.

## Methods

### Participants and Study Design

All study subjects provided written informed consent before participation in this study. The study protocols were approved by the medical ethics committees at Kyoto Prefectural University of Medicine (approval number: RBMR-C-559-5 and ERB-C-1702) and Hokkaido University Graduate School of Medicine (approval number: 14-004). Informed consent was obtained from each subject when possible, or from the appropriate legal guardian when not possible. All study procedures were designed and performed in accordance with the Declaration of Helsinki. According to a priori power analysis (with 80% power and 5% type I error rate) of the results of previous studies (9, 10), the minimum number for the sample was found to be 24 patients for the comparative study of the blood caffeine level in PD and control groups.

In the first cohort (discovery cohort), caffeine levels in serum were measured. Serum samples were collected from the registrations for dementia and related disorders in Kyoto Prefectural University of Medicine (KPUM) from September 2009 to March 2014. Clinical data, including Hoehn & Yahr (H&Y) stages, Unified Parkinson's Disease Rating Scale motor section (UPDRS-III) scores, heart/mediastinum (H/M) ratio in the early phases of myocardial imaging with 123I-metaiodobenzylguanidine (MIBG), and levodopa equivalent daily dose (LEDD) were evaluated within 1 month of sample collection. Plasma samples of the second cohort were collected at Hokkaido University from July 2008 to July 2018.

Patients were eligible for inclusion if they had been diagnosed according to the internationally standardized criteria of PD (11) and MSA (12). We enrolled age-matched participants with PD and controls in the first cohort as well as with PD, MSA, and controls in the second cohort. On the other hand, the age-matching between PD and MSA in the first cohort was incomplete because of an insufficient number of MSA patients. Subjects were excluded if they had a history of cancer, aspiration pneumonia, or collagen vascular diseases. Serum and plasma samples were obtained via venous puncture under resting conditions in the hospital. Subjects were asked not to eat anything or to drink caffeinated beverages for at least 3 h before sampling. After collection, serum and plasma were separated by centrifugation for 15 min at 2,000 g, then stored at −80°C until analysis.

### Caffeine ELISA

ELISA plates (96-well RIA/EIA Clear Flat Bottom Polystyrene High Bind Microplate; Corning Inc., Corning, NY) were coated by overnight incubation at 4°C with 2 μg/ml of anti-caffeine monoclonal antibody (CalBioReagents, San Mateo, CA) at 100 μl/well diluted in phosphate-buffered saline (PBS). Each plate was washed five times with PBS containing 0.05% Tween 20 (PBST) and incubated with blocking buffer (2% bovine serum albumin) for 30 min at 37°C. After washing five times with PBST, 50 μl of standard solutions (Caffeine OQ/PV sample; Agilent Technologies, Santa Clara, CA) or samples, and 50 μl of horseradish peroxidase-conjugated caffeine (CalBioReagents) was added to each well. After 30 min of incubation at 37°C, plates were washed, and 100 μl per well of substrate solution (1 step ultra TMB ELISA; Thermo Fisher Scientific, Rockford, IL) was added. After incubation for 15 min at room temperature in a dark room, color development was stopped with 100 μl/well of 1 mol/L sulfuric acid and absorbance was measured at 450 nm using a microplate spectrophotometer (SpectraMax Plus 384; Molecular Devices Corporation, Tokyo, Japan). For quantitation, standards were fitted to a logistic model curve. Both standards and samples were determined in triplicate on each plate.

### Validation of ELISA Methods

The procedure of method validation was conducted according to the guiding principles of the previous report (13). All experiments were performed using serum samples as well as plasma samples.

#### Precision

We collected two samples with known high and low concentrations of caffeine and made 20 aliquots of each sample. On days 1–4, we measured five replicates on each sample and calculated the mean, SD, and coefficient of variation (%CV) for both repeatability and intermediate precision.

#### Limits of Quantification and Detection

We prepared 16 aliquots of a blank sample (PBS solution), measured “background” signals by caffeine ELISA, and calculated the mean and SD of the signal. The lower limit of quantification (LOQ) and lower limit of detection (LOD) of the assay were determined as an interpolated caffeine concentration derived from signals of mean minus 10 SD and 2.5 SD of the value of signals for blank samples, respectively.

#### Dilution Linearity and Parallelism

Serial dilutions of three samples, which were diluted with PBS in small vials until the theoretical concentration was below the lower LOQ, were analyzed in duplicate, on the same plate and compensated for the dilution factor. For each sample, %CV was calculated using results from the dilutions.

#### Recovery

Three samples with determined concentrations of caffeine were collected. Three aliquots of the same sample were prepared and spiked with 0 and 100 μg or 200 μg of caffeine for spike recovery experiments. Recovery rates were calculated using the following formula:

$$\begin{array}{l} Recovery\;rate\;\left(\%\right)=\frac{Measured\;Concentration\;spiked\;sample-Measured\;Concentration\;neat\;sample}{Theoritical\;Concentration}\;\times \;100 \end{array}$$

### Concentration After Caffeine Intake

We measured caffeine levels for three healthy volunteers in their thirties before and 2 h after drinking commercially available coffee. They did not eat anything or drink caffeinated beverages for at least 3 h before first sampling. Two of the volunteers drank 2 mg/kg of caffeine and the other drank 4 mg/kg of caffeine.

### Statistical Analysis

Mean differences in caffeine between PD, MSA, and Control groups were analyzed by one-way ANOVA and Bonferroni's multiple comparison test. Regression lines between caffeine levels and clinical parameters were assessed using the Spearman rank correlation test. We also derived receiver operating characteristic (ROC) curves for the diagnosis of PD and MSA using caffeine levels as the predictor and estimated area under the curve (AUC; AUC = 0.5 indicates no discrimination, AUC = 1 indicates a perfect diagnostic test) to evaluate the diagnostic utility of caffeine. Significance was accepted for values of *p* < 0.05. Statistical analyses were performed using GraphPad Prism software (GraphPad, La Jolla, CA).

## Results

### Quality Performance of Caffeine ELISA

Intra-assay %CV, as a predictor of the precision of repeatability, was 13.4% in the sample with a low concentration of caffeine (621 ng/ml), and 15.1% in the sample with a high concentration of caffeine (7,914 ng/ml). Inter-assay %CV, as a predictor of immediate precision, was 12.1% in the former group and 15.1% in the latter group. Lower LOQ and LOD from caffeine ELISA were 3.66 and 0.35 ng/ml, respectively.

**Figure 1:**
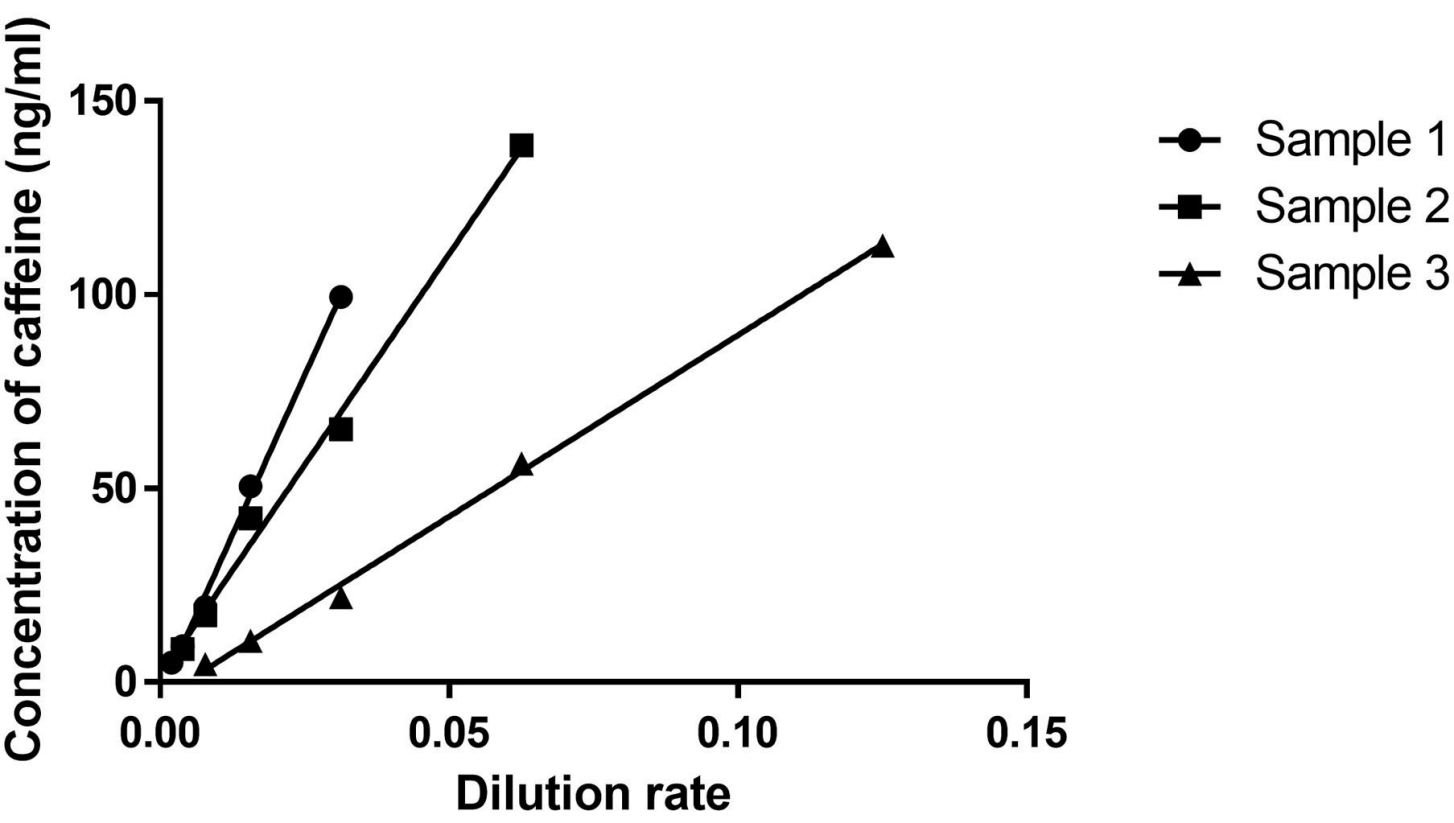
Dilutional linearity. After screening various control plasma samples, we chose three serum samples representing low (Sample 1), middle (Sample 2), and high (Sample 3) levels of caffeine. A serum dilution study was then undertaken to analyze linearity and slope in those three samples. X and Y axes indicate dilution rates and concentrations of caffeine (ng/ml).

Figure 1 shows dilution curves between the lower and upper LOQ of the assay based on the results of dilutional linearity and parallelism. Upper LOQ was estimated as 150 ng/ml. Goodness of fit was 0.99 on Sample 1, 0.98 on Sample 2, and 0.99 on Sample 3. The recovery rate of each sample was 87–113% (Supplementary Table 1).

Similar results were obtained in the experiments with plasma samples (Supplementary Table 2).

### Changes in Serum Caffeine Concentration With Coffee Intake

Caffeine concentration in serum increased with coffee intake in proportion to the volume of intake (Table 1). Consuming caffeine led to an 8- to 12-time elevation in serum caffeine signals on ELISA.

**Table 1:** Caffeine concentrations in serum samples before and after coffee intake.

	**Caffeine concentration in serum (ng/ml)**	
	**Before coffee intake**	**After coffee intake**
Sample 1	348	2,761
Sample 2	589	2,275
Sample 3	612	7,914

### Serum Concentrations of Caffeine in the First Cohort

**Figure 2:**
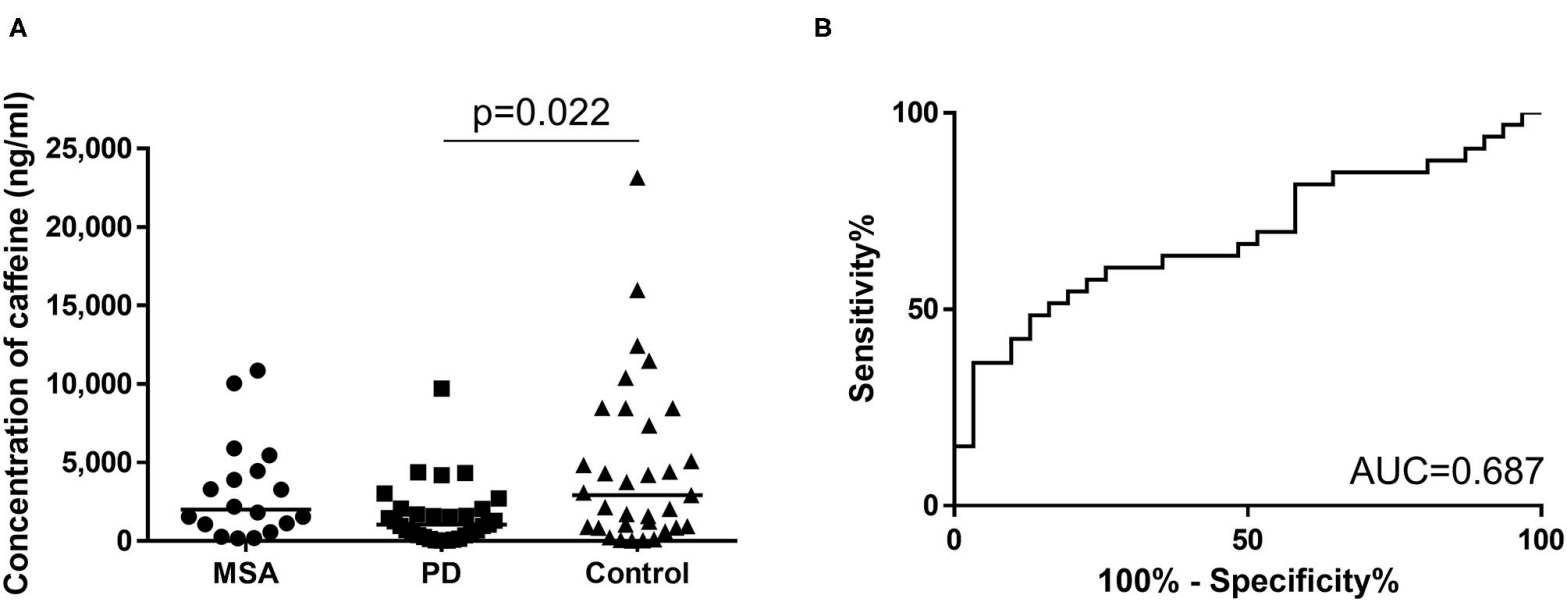
**(A)** Scatter plots of caffeine levels in the first cohort showing serum levels of caffeine in the MSA (*n* = 18), PD (*n* = 31), and control (*n* = 33) groups. Bars indicate median values. Levels of caffeine were significantly lower in the PD group than in the Control group. No significant difference in plasma caffeine levels was seen between PD and MSA groups (PD vs. MSA: *p* = 0.079). **(B)** Receiver operating characteristic (ROC) curves of caffeine for differential diagnosis of PD and Controls in the first cohort. Area under the curve (AUC) is 0.687.

We enrolled 82 subjects, comprising 31 patients with PD [mean (± SD) age, 65.2 ± 12.9 years; range, 41–84 years; 23 men, eight women] and 18 patients with MSA (mean age, 60.9 ± 8.3 years; range, 44–75 years; eight men, 11 women), and 33 age-matched disease controls (mean age, 61.5 ± 17.7 years; range, 16–84 years; 24 men, nine women). Disease controls included patients with cranial and peripheral neuropathy (*n* = 11), cervical spondylosis (*n* = 11), myopathy (*n* = 3), disuse syndrome (*n* = 2), benign positional vertigo (*n* = 1), idiopathic intracranial hypertension (*n* = 1), hyponatremia (*n* = 1), Asperger syndrome (*n* = 1), head drop syndrome (*n* = 1), and dysarthria (*n* = 1). The demographic data are shown in Supplementary Table 3. Caffeine concentrations in serum samples from the first cohort are summarized in Figure 2A significant overall difference among the three groups was observed from one-way ANOVA. *Post-hoc* analysis showed that serum concentrations were significantly lower in patients with PD compared to Controls, but only tended to be lower in the MSA group compared to Controls, with no significant difference (PD vs. Control: *p* = 0.022; MSA vs. Control: *p* > 0.999; PD 1,608 ± 1,966 ng/mL, MSA 3,212 ± 3,172 ng/mL, Control 4,650 ± 5,308 ng/mL; Figure 2A). The difference between PD and MSA groups was not significant (PD vs. MSA, *p* = 0.079). The AUC of ROC curves for the classification of patients with PD and disease controls was 0.687 (Figure 2B).

### Plasma Concentrations of Caffeine in the Second Cohort

**Figure 3:**
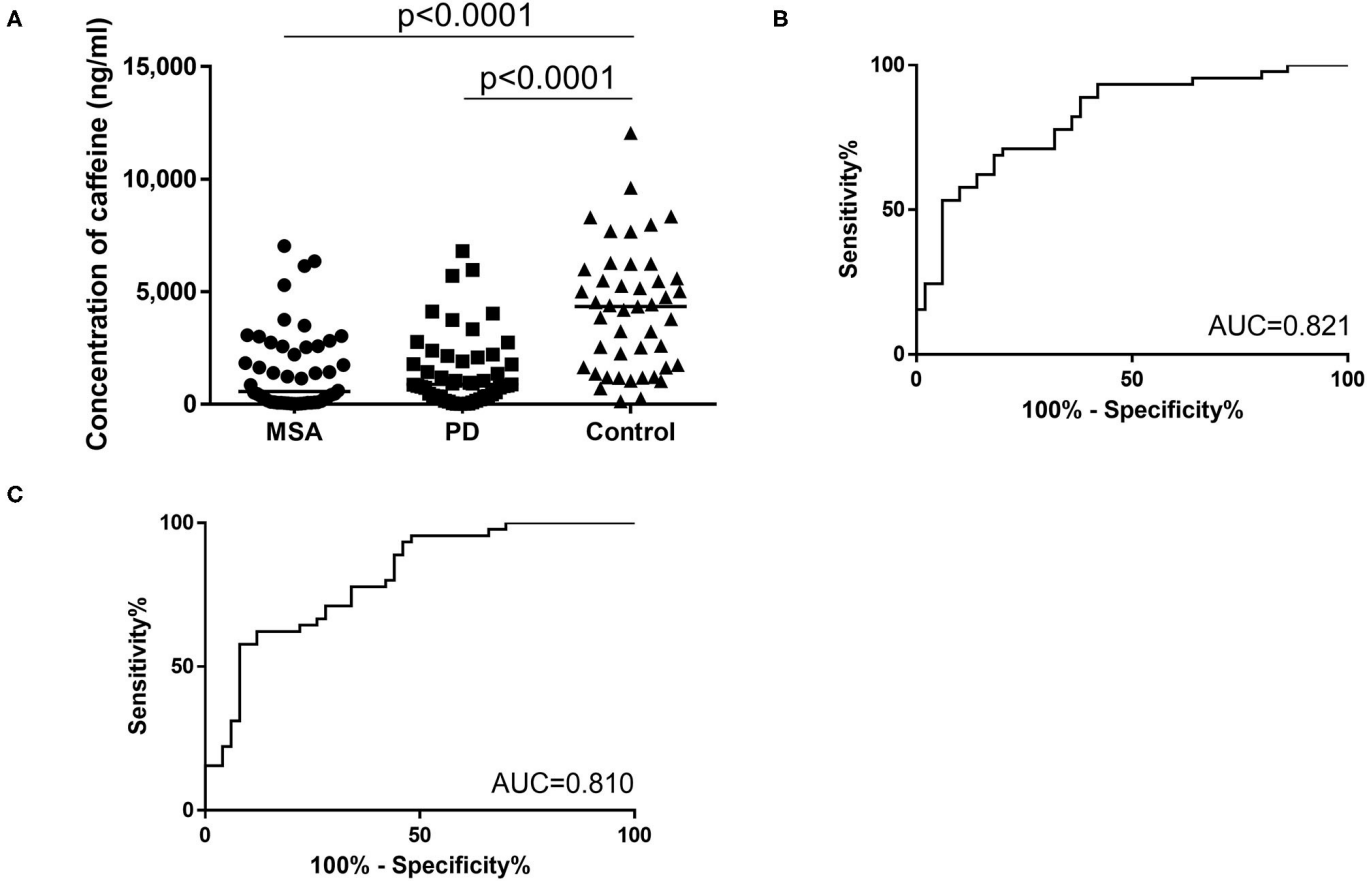
**(A)** Scatter plots of caffeine levels in the second cohort showing levels of plasma caffeine in the MSA (*n* = 50), PD (*n* = 50), and control (*n* = 45) groups. Bars indicate median values. Levels of caffeine were significantly lower in the PD and MSA groups than in the Control group. Plasma caffeine levels did not differ significantly between PD and MSA groups (MSA vs. PD: *p* > 0.999). **(B,C)** Receiver operating characteristic (ROC) curves of caffeine for differential diagnosis of PD and Controls **(B)** and for differential diagnosis of MSA and controls **(C)** in the second cohort. Area under the curve (AUC) is 0.821 for PD and controls and 0.810 for MSA and controls.

The second cohort comprised 50 patients with PD (mean age, 71.5 ± 6.9 years; range, 53–84 years; 25 men, 25 women), 50 patients with MSA (mean age, 68.6 ± 7.0 years; range, 53–81 years; 25 men, 25 women) and 45 age-matched healthy controls (mean age, 70.4 ± 7.0 years; range, 56–80 years; 25 men, 25 women). As with the first cohort, a significant overall difference was seen between groups. Plasma caffeine concentrations were significantly lower in patients with PD than in Controls (PD vs. Control, *p* < 0.001; MSA vs. Control, *p* < 0.001; PD 1,459 ± 1,616 ng/mL, MSA 1,484 ± 1,805 ng/mL, Ctrl 4,186 ± 2,740 ng/mL; Figure 3A) in *post-hoc* tests. Moreover, plasma concentrations of caffeine in patients with MSA were significantly decreased compared to Controls, and did not differ significantly from those in PD (MSA vs. PD: *p* > 0.999). AUCs of ROC curves for patients with PD and MSA compared with disease controls were 0.821 and 0.810, respectively (Figures 3B,C).

### Correlation Between Caffeine Level and Clinical Characteristics of Patients With PD and MSA

Serum caffeine levels in the PD group did not correlate with H&Y stage, UPDRS-III score, or H/M ratio of MIBG uptake (Supplementary Figures 1A–D). Serum caffeine levels in the PD group did not correlate with duration from onset (Supplementary Figure 1E). No significant correlation was identified between serum caffeine levels and age in the PD, MSA, and Control groups (Supplementary Figures 1F–H).

## Discussion

The originally developed ELISA examined in this study offered sufficiently high sensitivity to detect caffeine in human serum and plasma. Both intra- and inter-assay precision (%CV) of our assay were below 20%, which is the recommended acceptance criterion for single-laboratory accuracy of a biomarker (14). Consuming caffeine led to an 8- to 12-time elevation in serum caffeine signals on ELISA. This rate of increase supports the findings of a previous observation (15), suggesting that this caffeine ELISA system reasonably worked even *in vivo*.

The assays in the first cohort showed a significant decrease in serum caffeine concentration for PD patients as compared to Controls. This result was reproducibly observed in plasma levels of caffeine in the second cohort. Those findings were consistent with previous metabolomic analyses (8, 9) and also matched with a report stating that serum concentrations of the caffeine metabolite, theophylline, were significantly decreased in the PD group compared to controls (10). In the second cohort, plasma caffeine concentration of MSA was significantly decreased compared to that of the control group. A similar trend was observed in the first cohort despite the lack of significance. This result implied that plasma and serum caffeine levels in MSA might be lower than those in controls, similar to the case of PD. Fujimaki et al. speculated that impaired gastrointestinal dysfunction and dysmotility in PD were responsible for reduced caffeine levels, based on the lack of correlation between caffeine intake and blood caffeine levels in their PD cohort. The decreased plasma caffeine levels in MSA, which also cause serious autonomic failure including intestinal dysfunction, could support this speculation.

Serum caffeine levels in the PD group of the first cohort did not correlate with age, duration from onset, severity of disease, or H/M ratio of MIBG uptake. These results are consistent with those of previous reports (8, 9).

We acknowledge that the small sample size represents a major limitation to this study. As other limitations, no information was available regarding the daily caffeine intakes or genetic backgrounds of participants. In the future, case-control studies involving sufficient numbers of participants with information on caffeine consumption as well as genetic information affecting caffeine pharmacokinetics (e.g., ADORA2A and CYP1A2 gene polymorphisms) are needed to confirm our findings (16). Moreover, it remains unclear in the current study why PD (and MSA) patients have lower blood caffeine levels. It might be because of less consumption, less absorption in the gastrointestinal tract, or quicker metabolism of caffeine. To test these hypothetical ideas, comparison of time-dependent changes of blood caffeine levels after caffeine ingestion between PD/MSA patients and controls would be also important in future studies.

## Conclusion

We have developed a specific ELISA system for detecting caffeine in blood and performed method validation. This originally developed ELISA offered sufficiently high sensitivity to detect caffeine in human serum and plasma. We reproducibly confirmed decreased blood caffeine concentrations in PD compared to controls by ELISA. These findings confirm previous observations from retrospective case-control studies, and provide evidence that this ELISA can work as a diagnostic biomarker for PD. A similar decreasing trend was also observed in the MSA group. This result implies potential clinical utility of blood caffeine levels as a biomarker for MSA. Future validation studies are still needed for this issue, because of a lack of consistent significant differences across cohorts.

## Data Availability Statement

The original contributions presented in the study are included in the article/Supplementary Materials, further inquiries can be directed to the corresponding author/s.

## Ethics Statement

The studies involving human participants were reviewed and approved by Medical Ethics Committees at Kyoto Prefectural University of Medicine and Hokkaido University Graduate School of Medicine. The patients/participants provided their written informed consent to participate in this study.

## Author Contributions

TKa and TT: conception and study design. TO, HT, FK-M, MS, JM, TKo, and HS: data acquisition. TO and TKa: drafting first version of the manuscript and figure and tables. TO, TKa, MS, JM, TKo, FK-M, HT, HS, TM, and TT: final version of the manuscript. TM: study supervision. All authors contributed to the article and approved the submitted version.

## Conflict of Interest

The authors declare that the research was conducted in the absence of any commercial or financial relationships that could be construed as a potential conflict of interest.

## References

[1] Schwarzschild MA Xu K Oztas E Petzer JP Castagnoli K Castagnoli N. Neuroprotection by caffeine and more specific A2A receptor antagonists in animal models of Parkinson's disease. Neurology. (2003) 61(11 Suppl. 6):S55–61. 10.1212/01.WNL.0000095214.53646.72 14663012

[2] Noyce AJ Bestwick JP Silveira-Moriyama L Hawkes CH Giovannoni G Lees AJ. Meta-analysis of early nonmotor features and risk factors for Parkinson disease. Ann Neurol. (2012) 72:893–901. 10.1002/ana.23687 23071076 PMC3556649

[3] Ascherio A Chen H. Caffeinated clues from epidemiology of Parkinson's disease. Neurology. (2003) 61(11 Suppl. 6):S51–4. 10.1212/01.WNL.0000095213.86899.21 14663011

[4] Qi H Li S. Dose-response meta-analysis on coffee, tea and caffeine consumption with risk of Parkinson's disease. Geriatr Gerontol Int. (2014) 14:430–9. 10.1111/ggi.12123 23879665

[5] Chen JF Xu K Petzer JP Staal R Xu YH Beilstein M. Neuroprotection by caffeine and A(2A) adenosine receptor inactivation in a model of Parkinson's disease. J Neurosci. (2001) 21:Rc143. 10.1523/JNEUROSCI.21-10-j0001.2001 11319241 PMC6762498

[6] Mizuno Y Kondo T. Adenosine A2A receptor antagonist istradefylline reduces daily OFF time in Parkinson's disease. Mov Disord. (2013) 28:1138–41. 10.1002/mds.25418 23483627 PMC3842830

[7] LeWitt PA Li J Lu M Guo L Auinger P. Metabolomic biomarkers as strong correlates of Parkinson disease progression. Neurology. (2017) 88:862–9. 10.1212/WNL.0000000000003663 28179471 PMC5331866

[8] Hatano T Saiki S Okuzumi A Mohney RP Hattori N. Identification of novel biomarkers for Parkinson's disease by metabolomic technologies. J Neurol Neurosurg Psychiatry. (2016) 87:295–301. 10.1136/jnnp-2014-309676 25795009

[9] Fujimaki M Saiki S Li Y Kaga N Taka H Hatano T. Serum caffeine and metabolites are reliable biomarkers of early Parkinson disease. Neurology. (2018) 90:e404–11. 10.1212/WNL.0000000000004888 29298852 PMC5791797

[10] Ohmichi T Kasai T Kosaka T Shikata K Tatebe H Ishii R. Biomarker repurposing: therapeutic drug monitoring of serum theophylline offers a potential diagnostic biomarker of Parkinson's disease. PLoS ONE. (2018) 13:e0201260. 10.1371/journal.pone.0201260 30044870 PMC6059449

[11] Hughes AJ Daniel SE Kilford L Lees AJ. Accuracy of clinical diagnosis of idiopathic Parkinson's disease: a clinico-pathological study of 100 cases. J Neurol Neurosurg Psychiatry. (1992) 55:181–4. 10.1136/jnnp.55.3.181 1564476 PMC1014720

[12] Gilman S Wenning GK Low PA Brooks DJ Mathias CJ Trojanowski JQ. Second consensus statement on the diagnosis of multiple system atrophy. Neurology. (2008) 71:670–6. 10.1212/01.wnl.0000324625.00404.15 18725592 PMC2676993

[13] Andreasson U Perret-Liaudet A van Waalwijk van Doorn LJ Blennow K Chiasserini D Engelborghs S. A practical guide to immunoassay method validation. Front Neurol. (2015) 6:179. 10.3389/fneur.2015.00179 26347708 PMC4541289

[14] DeSilva B Smith W Weiner R Kelley M Smolec J Lee B. Recommendations for the bioanalytical method validation of ligand-binding assays to support pharmacokinetic assessments of macromolecules. Pharm Res. (2003) 20:1885–900. 10.1023/B:PHAM.0000003390.51761.3d 14661937

[15] Chen F Hu ZY Parker RB Laizure SC. Measurement of caffeine and its three primary metabolites in human plasma by HPLC-ESI-MS/MS and clinical application. Biomed Chromatogr. (2017) 31:e3900. 10.1002/bmc.3900 27864843 PMC5415443

[16] Popat RA Van Den Eeden SK Tanner CM Kamel F Umbach DM Marder K. Coffee, ADORA2A, and CYP1A2: the caffeine connection in Parkinson's disease. Eur J Neurol. (2011) 18:756–65. 10.1111/j.1468-1331.2011.03353.x 21281405 PMC3556904

