# Supplementary material for: Quantification of Blood Caffeine Levels in Patients With Parkinson's Disease and Multiple System Atrophy by Caffeine ELISA
Source: Front Neurol. 2020 Dec 22;11:580127. doi: 10.3389/fneur.2020.580127 (PMC7783046; doi:10.3389/fneur.2020.580127)
Supplement: Supplementary file 1 [file Data_Sheet_1.docx]

Supplementary Material

##
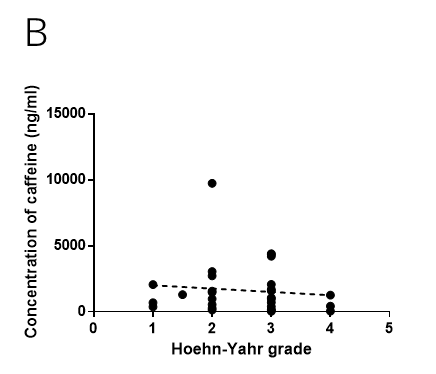

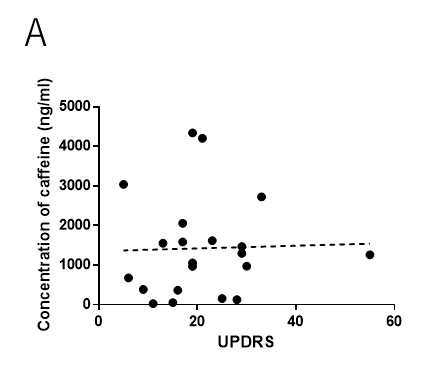
1 Supplementary Figures


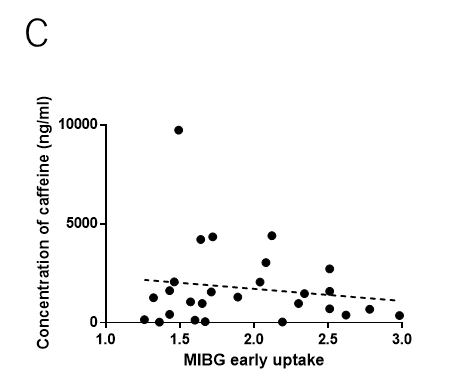

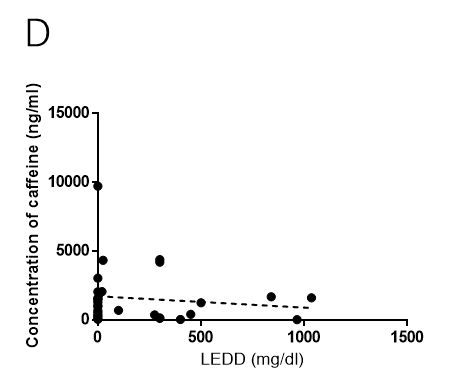


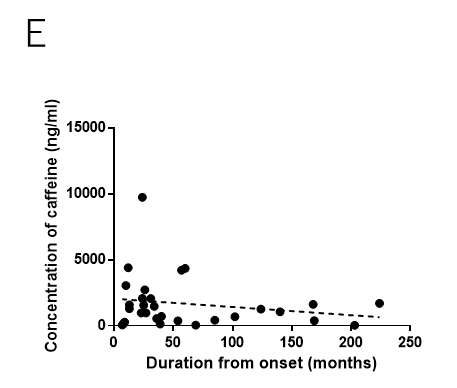

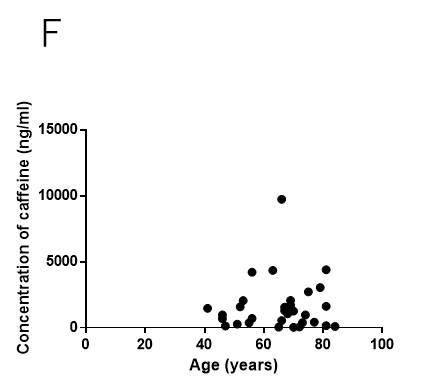


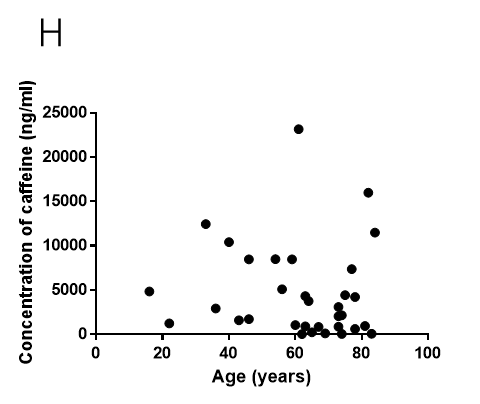

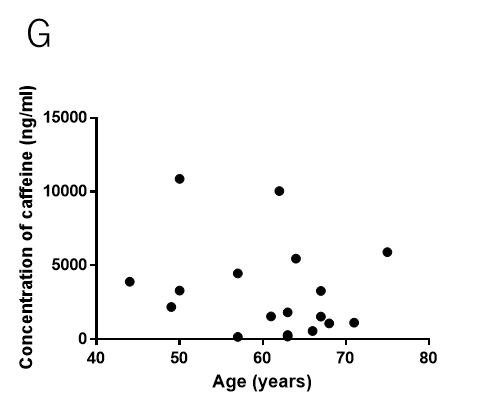


**Supplementary Figure 1.** Associations between serum levels of caffeine and UPDRS-III score (A), H&Y stage (B), H/M ratio in the early phase on MIBG myocardial images (C), levodopa equivalent daily dose (D) and duration from onset (E) in the PD group. Associations between serum levels of caffeine and age in PD (F), MSA (G) and control (H) groups. No significant correlations are evident.

**2 Supplementary Tables**

**Supplementary Table 1**

The recovery rate for each sample in quality performance of caffeine ELISA in serum.

|  | Theoretical concentration of spike (ng/ml) | Mean concentration (ng/ml) | CV (%) | Recovery rate (%) |
| --- | --- | --- | --- | --- |
| Sample 1 | 0 | 13.7 | 3.8 | 0 |
|  | 5 | 18.965 | 10.5 | 105 |
|  | 10 | 23.843 | 17.7 | 101 |
| Sample 2 | 0 | 9.6 | 1.6 | 0 |
|  | 5 | 13.9 | 0.2 | 87 |
|  | 10 | 20.9 | 7.4 | 113 |
| Sample 3 | 0 | 3.9 | 15.1 | 0 |
|  | 5 | 8.9 | 2.6 | 100 |
|  | 10 | 13.4 | 11.7 | 95 |

CV = coefficient of variation

**Supplementary Table 2**

Quality performance of caffeine ELISA in plasma

Intra-assay %CV, as a predictor of repeatability precision, was 11.4% in the sample with a low concentration of caffeine (821 ng/ml), and 18.1% in the sample with a high concentration of caffeine (7914 ng/ml). Inter-assay %CV, as a predictor of immediate precision, was 11.4% in the former and 11.2% in the latter.

Goodness of fit was 0.97 on Sample 1, 0.95 on Sample 2, and 0.99 on Sample 3. The recovery rate for each sample ranged from 78% to 118%.

|  | Theoretical concentration of spike (ng/ml) | Mean concentration (ng/ml) | CV (%) | Recovery rate (%) |
| --- | --- | --- | --- | --- |
| Sample 1 | 0 | 5.0 | 16.3 | 0 |
|  | 10 | 16.8 | 16.1 | 118 |
| Sample 2 | 0 | 5.0 | 2.9 | 0 |
|  | 10 | 12.9 | 1.4 | 78 |
| Sample 3 | 0 | 40.5 |  | 0 |
|  | 10 | 51.9 | 5.8 | 115 |

CV = coefficient of variation

**Supplementary Table 3**

Characteristics of patients with PD, MSA and disease controls in the first cohort

Data for continuous variables are expressed as median values (maximum-minimum). The rows of the

H&Y stage indicate numbers of patients in each grade. Percentages of the subjects in each group are

presented in brackets.

|  | PD group (n=31) | MSA group (n=19) | Control group (n=33) |
| --- | --- | --- | --- |
| Age (years) | 65 (41-81) | 61 (44-75) | 62 (16-84) |
| Sex (numbers of females) | 8 [26%] | 11 [58%] | 9 [27%] |
| H&Y stage |  |  |  |
| Grade 1 | 4 [13%] | - | - |
| Grade 2 | 10 [32%] | - | - |
| Grade 3 | 14 [45%] | - | - |
| Grade 4 | 3 [10%] | - | - |
| UPDRS-III (points) | 19 (5-55) | - | - |
| Duration from onset (months) | 35 (8-224) | - | - |
| H/M ratio of MIBG |  |  |  |
| Early phase | 1.81 (1.26-2.98) | - | - |
| Delayed phase | 1.46 (1.14-3.24) | - | - |
| MMSE (points) | 29 (19-30) | - | - |
| LEDD (mg/day) | 0 (0-1035) | 0 (0-200) | - |

PD = Parkinson’s disease, MSA = multiple system atrophy, H&Y = Hoehn & Yahr, UPDRS-III = Unified Parkinson’s Disease Rating Scale motor section, H/M = heart/mediastinum (H/M), MIBG = myocardial imaging with 123I-metaiodobenzylguanidine, LEDD = levodopa equivalent daily dose
